# Early Stage Biomineralization in the Periostracum of the ‘Living Fossil’ Bivalve *Neotrigonia*

**DOI:** 10.1371/journal.pone.0090033

**Published:** 2014-02-25

**Authors:** Antonio G. Checa, Carmen Salas, Elizabeth M. Harper, Juan de Dios Bueno-Pérez

**Affiliations:** 1 Departamento de Estratigrafía y Paleontología, Facultad de Ciencias, Universidad de Granada, Granada, Spain; 2 Departamento de Biología Animal, Facultad de Ciencias, Universidad de Málaga, Málaga, Spain; 3 Department of Earth Sciences, University of Cambridge, Cambridge, United Kingdom; 4 Centro de Instrumentación Científica, Universidad de Granada, Granada, Spain; Institute for Frontier Medical Sciences, Kyoto University, Japan

## Abstract

A detailed investigation of the shell formation of the palaeoheterodont ‘living fossil’ *Neotrigonia* concentrated on the timing and manufacture of the calcified ‘bosses’ which stud the outside of all trigonioid bivalves (extant and fossil) has been conducted. Electron microscopy and optical microscopy revealed that *Neotrigonia* spp. have a spiral-shaped periostracal groove. The periostracum itself is secreted by the basal cell, as a thin dark pellicle, becoming progressively transformed into a thin dark layer by additions of secretions from the internal outer mantle fold. Later, intense secretion of the internal surface of the outer mantle fold forms a translucent layer, which becomes transformed by tanning into a dark layer. The initiation of calcified bosses occurred at a very early stage of periostracum formation, deep within the periostracal groove immediately below the initialmost dark layer. At this stage, they consist of a series of polycyclically twinned crystals. The bosses grow as the periostracum traverse through the periostracal groove, in coordination with the thickening of the dark periostracal layer and until, upon reaching the mantle edge, they impinge upon each other and become transformed into large prisms separated by dark periostracal walls. In conclusion, the initial bosses and the external part of the prismatic layer are fully intraperiostracal. With later growth, the prisms transform into fibrous aggregates, although the details of the process are unknown. This reinforces the relationships with other groups that have the ability to form intraperiostracal calcifications, for example the unionoids with which the trigonioids form the clade Paleoheterodonta. The presence of similar structures in anomalodesmatans and other euheterodonts raises the question of whether this indicates a relationship or represents a convergence. The identification of very early calcification within an organic sheet has interesting implications for our understanding of how shells may have evolved.

## Introduction

Molluscan shells are biocomposites formed by highly ordered calcium carbonate crystals in an organic matrix. The way in which molluscs make their shells has been of great interest for a variety of reasons; because of their advantageous mechanical properties, understanding their formation is of interests in the search for novel synthetic materials [1], [2] and biomedicine application [3]–[6], while the arrangement of different microstructures within their shells has been shown to have significance in the study of phylogenetic relationships [7]–[9] and of the adaptive significance of different microstructures, e.g. [10]–[12], within the phylum. The majority of research has focussed on the calcium carbonate structures of the shell [13]–[16] but more recently new researches have focussed on the role of the organic macromolecules in the formation and biomineral assembly [17]–[20]. Relatively few studies, however, have investigated the role of the predominantly organic periostracum, an outer shell layer which is secreted by the mantle before the calcareous part of the shell and which has a fundamental importance in shell manufacture, in both isolating the site of biomineralization from the contaminating effects of sea-water and providing the template onto which the mineralization process of the shell is carried out [21]–[24]. The periostracum is also important for protecting the shell from dissolution in corrosive environments [25] or biological attack [26], [27]. Although the periostracum is usually considered as an unmineralised conchiolin (proteinaceous) layer of the shell [13], periostracal calcification has been reported in a number of taxa [24], [28]–[36]. Although a number of different clades of bivalves are known to calcify parts of their periostraca, there seems to be a particular propensity within the Palaeoheterodonta and Heterodonta (and in particular the more basal taxa). The varied nature of the calcification suggests perhaps that these are not synapomorphic characters that link particular taxa but rather reflect a deeper homology in the possession of some characters within the periostraca that facilitate calcification [34], while other authors [28], [37] have suggested that these structures may represent a symplesiomorphic bivalve condition.

In a series of papers we have investigated the occurrence, characteristics and formation of periostracal calcification within the palaeoheterodont and heterodont bivalves [34]–[36]. Within this group of taxa, the palaeoheterodont trigonioid bivalves have not been examined in detail. The present study focuses on the origin and formation of the periostracum in the trigonioid bivalve *Neotrigonia,* in particular on the morphological and histological study of the development changes of the periostracum from its formation in the periostracal groove to its complete calcification at the shell margin. The key finding is the demonstration of calcification within the periostracum at a very early stage inside the periostracal groove.

### Neotrigonia

The palaeoheterodont genus *Neotrigonia* is the only remaining genus of the Trigonioida, an order which dates back until the early Palaeozoic [38]. Although members of this order were conspicuous and abundant components of shallow marine communities throughout the Mesozoic world-wide, they suffered major losses at the end Cretaceous mass extinction. The only survivors, which might be considered as “living fossils” [39], [40], were confined to the Australasian and southwest Pacific region waters [41], [42]) where today they are represented by eight species (WoRMS 2014, January 30^th^), *N. margaritacea* (Lamarck, 1804), *N. lamarckii* (Gray, 1838), *N. uniophora* (Gray in Jukes, 1847), *N. strangei* (A. Adams, 1854), *N. bednalli* (Verco, 1907), *N. gemma* Iredale, 1924, *N. kaiyomaruae* Habe & Nomoto, 1976 and *N. jacksoni* Morrison, 2011. However *N. bednalli* has been questioned as valid species and it is proposed as junior synonym of *N. margaritacea* [43], [44]. Aside from their interest as ‘living fossils”, they are also phylogenetically interesting because of their position within the Palaeoheterodonta, as the sister-group to one of the major freshwater clades, the Unionoida [45]–[47,]. The Palaeoheterodonta have traditionally been regarded as the sister-group of the Heterodonta, though more recent molecular analyses [47], [48]) now support the relationship between the Palaeoheterodonta and the Archiheterodonta.

**Figure 1:**
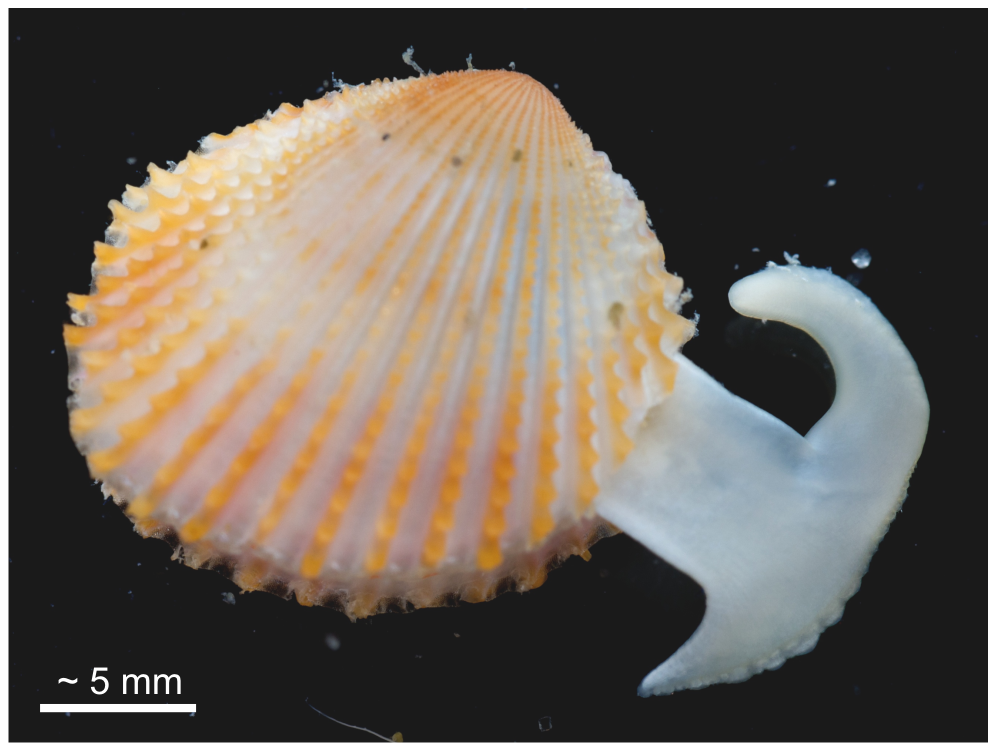
Living specimen of *Neotrigonia lamarckii*, with the extended foot.

All modern and fossil trigoniids have sub-quadrate, trigonal equivalve and relatively thick shells, with radial ribs frequently adorned by tubercles or scales (Fig.1); the valves are articulated with a relatively massive schizodont dentition. In spite of their highly ornamented shells, modern *Neotrigonia* are moderately rapid burrowers using a large muscular L-shaped foot with a distinctive ‘toe’ and ‘heel’ (Fig. 1) which is also used to execute vigorous leaping as an escape mechanism from predators [39], [49], [50]. All species are subtidal, living in sediments ranging from poorly sorted sand to sandy mud [51].

The shell of *Neotrigonia* has been described as being wholly aragonitic, with an outer prismatic layer and inner layers of lenticular ( = columnar) and sheet nacre [7], [52]. An interesting feature of the outer surface of the shell is that each prism in the outer layer begins with a ‘boss’ which is embedded within the periostracum, features which are evident even in fossil material dating back to the Palaeozoic [53]. Taylor et al. [7] hypothesized that these bosses represent the initial spherulite “from which each prism has developed, on the inner surface of the periostracum”. Initial observations by scanning electron microscopy of the outer surface of shells revealed that the bosses appear free of periostracum and so may appear to have pierced through the periostracal layer [36] but it is not clear that these protrusions were not initially covered by a pellicle-like outer periostracum, which led these authors to suggest that they might initially grow within the periostracum, thus being intraperiostracal. It is, therefore, interesting to examine whether these calcified bosses are homologous to the calcified spikes reported in anomalodesmatans. Our specific aims were to carry out a study of the growth and structure of the periostracum in the genus *Neotrigonia* from its very early formation in the periostracal groove to its complete calcification at the shell margins and to investigate the precise relationship between the calcified bosses and the periostracum and the underlying prisms.

## Materials and Methods

### Ethics Statement

Ian Loch (The Australian Museum, Sidney) eased the loan of specimens of *Neotrigonia* listed in Table 1 under repository label AMS. Professor Gonzalo Giribet (Harvard University) and the BIVAToL project supported by the U.S. National Science Foundation (NSF) Assembling the Tree of Life (AToL) program (DEB-0732854/0732903/0732860) provided access to material and gave permission to use the image in Figure 1 (photographed by Professor Gonzalo Giribet). The specimen was collected in Moreton Bay Marine Park under Permit number QS2005/CVL588 from Queensland Parks and Wildlife Service. Dr. Ana Glavinic (School of Biological Sciences, Flinders University, Adelaide, Australia) sampled and provided the glutaraldehyde-fixed specimen of *N. margaritacea* from Port Stanvac, under the permit that the School of Biological Sciences has for such work (an S115 Ministerial Exemption Number 9002462) issued by the Primary Industries and Regions SA, Government of South Australia. No specific permission was required since the area is unprotected and the species is neither endangered nor protected. The loans and cessions of specimens were made under CITES guidelines, to which all the involved institutions are adhered. Professors Jan Bonarski and Marek Faryna, and Dr Katarzyna Berent (Institute of Metallurgy and Materials Science, Kraków, Poland) gave permission for the use of the crystallographic data shown in Figure 3F–I, acquired jointly with A. Checa.

**Table 1:** Collection details and localities for the material studied.

Species	Repository	N^1^	Locality	Fixative	Length^2^ (mm)
*N. gemma*	AMS^3^ C.73050	8	Loc #042655 NSW: off Cronulla, Sidney(34°4’S, 151°14’S)	5% saltwaterformalin	10.3; 14.6; 16.5
*N. lamarckii*	AMS C.313607	2	Loc #021996 NSW: Sidney (33°53.000’S,151°13.000’E)	70% EtOH	20.2
*N. margaritacea*	AMS C.363610	2	Loc #0042671 NSW: 10 km E of Cape Banks,Sidney (33°59.500’S, 151°20.500’E)	5% saltwaterformalin	17.4
	AMS C.413365	2	Loc #54151 SA: off Ceduna (32°24.000’S,133°30.000’E)	5% saltwaterformalin	38.5
	P/UGR^4^	1	Port Stanvac, Adelaide, South Australia	2.5%glutaraldehyde	22.5
	P/UGR	2	Anxious Bay (S. Australia)	Dry	23.7; 27.6
	P/UGR	1	Near Adelaide (S Australia)	Dry	36.5
	P/UGR	1	Coffin Bay (S Australia)	Dry	
	P/UGR	1	Loc. unknown	Dry	
*N. strangei*	AMS C.413366	2	Loc #2404 WA: North West Shelf, NNE of PortHedland (19°55.200’S-19°55.600’S, 117°56.000’E–117°55.600’S)	5% saltwaterformalin	10.8; 17.5
*N. uniophora*	AMS C.413364	2	Loc #000677 QLD: off Moreton Bay(27°27.370’S, 153°39.000’E)	5% saltwaterformalin	20.0

1 Number of specimens studied (all with paired valves);

2 Only specimens intact after the study;

3 The Australian Museum (Sidney);

4 Paleontology, University of Granada.

### Material and Techniques

The material studied consisted of a series of *in vivo* fixed specimens obtained from two sources (see Table 1 for collection details and localities). Most of the specimens were supplied on loan from The Australian Museum (Sidney) but because these were sub-optimally fixed for use in histological studies, we also obtained an individual specimen of *Neotrigonia margaritacea* from Port Stanvac, Adelaide, South Australia, fixed in 2.5% glutaraldehyde specifically for this study. This was also supplemented by the addition of dry valves from our own collections. The glutaraldehyde fixed specimen of *N. margaritacea,* as well as the dry shells, is presently housed at the paleontological collection of the University of Granada.

This suite of material has enabled us to undertake a scanning electron microscope (SEM) study of the shell microstructure of the 5 of the 8 extant species of *Neotrigonia* (Table 1). Microstructural details were assessed from fractures through different shell areas. Fragments were cleaned for 15 s in an ultrasonic bath and allowed to air dry before SEM (field emission SEM Zeiss LEO Gemini 1530 of the University of Granada) observation after carbon coating (Hitachi UHS evaporator). Additionally the mantle margin of one specimen of each of the species *N. margaritacea*, *N. lamarckii* and *N. gemma* were analysed by transmission electron microscopy (TEM) and optical microscopy (OM). Blocks of mantle, with their associated periostraca, were excised mainly from the ventral areas. The specimens were wholly decalcified by immersion in 2% EDTA for several days before post-fixation in OsO_4_ (2%) for 2 h at 4°C, and embedded in epoxy resin Aname Epon 812 (EMS). Semithin sections (1 µm) were stained with different concentrations of toluidine at pH 4.5 in order to better check the characteristics of the vesicles present in the epithelial cells of the outer mantle fold. Sections of *Neotrigonia margaritacea* were stained with: a) 1% toluidine blue in water with 1% Na_2_B_4_O_7_.10H_2_O (Merck 6308) at pH 4.5, which gives an intense blue stain; b) 1% toluidine blue in water with 0.2% acetic acid, at pH 4.5; c) 0.5% toluidine blue in water with 0.2% acetic acid, at pH 4.5; d) 0.2% toluidine blue in water with 0.2% acetic acid, at pH 4.5. Sections were observed with an Olympus BX51 optical microscope. Ultrathin sections (∼ 50 nm) were stained with uranyl acetate (1%) followed by lead citrate. They were observed with TEM (Zeiss EM10C and Zeiss Libra 120 Plus at the University of Granada). Pieces of the mantle margin, removed from different regions, of the glutaraldehyde-fixed specimen of *N. margaritacea* were also examined by SEM, for which they were critical-point dried, sputter-coated with gold and observed with the SEM JEOL JSM-840 of the University of Málaga.

The presence of Ca^2+^ in the epithelial cells of the outer mantle fold was confirmed by applying a fluorescent indicator (Fluo 3-AM, Sigma 46393) to semithin sections of *Neotrigonia margaritacea*. To obtain the stock solution, 1 mg of Fluo 3-AM was solubilized in 1 ml of a a buffer solution composed of 25% of Pluronic® F-127 (Sigma P2443) in dry dimethyl sulphoxide (DMSO) (Sigma 276855). In order to load the semithin sections, a work solution was prepared with 1 µl of the stock solution in 20 µl of buffer solution. To eliminate possible autofluorescence of the glutaraldehyde and the epoxy resin, the semithin sections were photobleached by exposing them to light for an extended period of time (∼1 h) [54]. For loading with Fluo 3-AM, the semithin sections were incubated for 1 h at room temperature (22–23°C). The fluorescence images were obtained with a laser confocal microscope (Leica DMI6000) with excitation by argon-ion laser source at 488 nm and maximum emission wavelength at 526 nm.

The crystallography of the prismatic units was analyzed by means of Electron Backscatter Diffraction (EBSD). Samples of *Neotrigonia margaritacea* were sectioned approximately parallel to the long axes of the prisms and polished on horizontal diamond-impregnated discs (Struers DP-U2 type polisher) with grit sizes 1 and 0.25 µm. Preparation was carried out at the Institute of Metallurgy and Materials Science (IMIM) of the Polish Academy of Sciences in Kraków. We used two sets of equipment. First, we used an Inca Crystal (Oxford Instruments) detector coupled to an Auriga Cross-Beam Station (Carl Zeiss) FESEM (Centro de Instrumentación Científica, Universidad de Granada). To avoid excessive charging, samples were coated with a thickness of 2 nm of carbon in a Baltec MED 020 electron beam evaporator. The second set of equipment (used for most analyses) was a TSL OIM detector coupled to FEI Field Emission Gun (FEG) SEM Quanta 3D microscope of the IMIM. Operation in low vacuum mode made coating unnecessary. A special cone was attached to the SEM pole piece to minimize the so-called “skirt effect” of the primary electron beam and reduce the gas-path length. Analysis software (TSL OIM version 5.3) was used to post-process the EBSD measurements. All data with a confidence index (CI) below 0.1 were removed. For visualization purposes the following cleanup procedure was applied: 1) grain CI standardization, 2) neighbor orientation correlation, 3) neighbor CI correlation.

## Results

### Shell Microstructure

**Figure 2:**
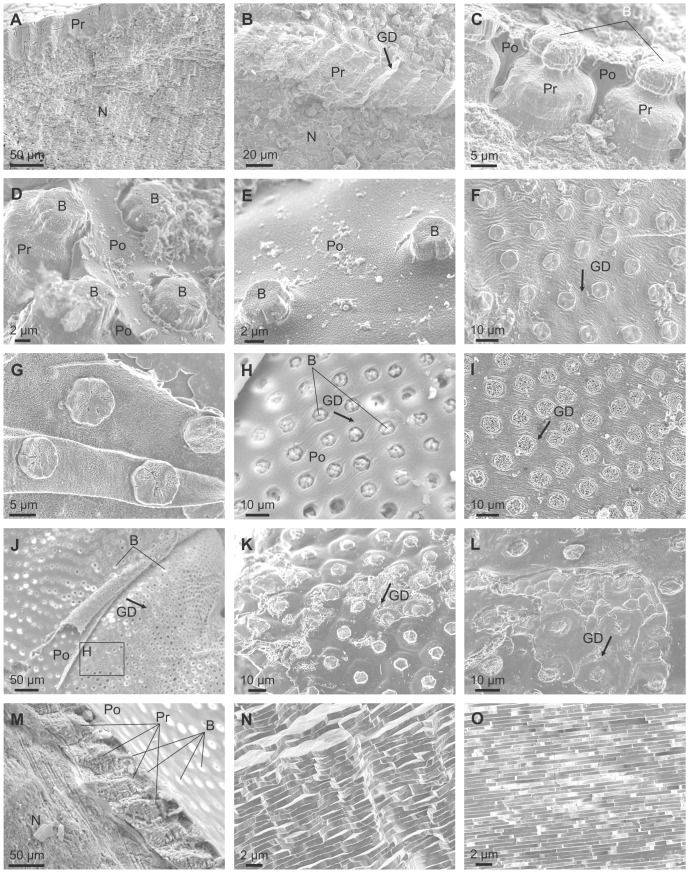
Shell microstructures of *Neotrigonia*. A. Vertical fracture through the prismatic and nacre layers of *N. lamarckii*. B. Fracture through the shell of *N. lamarckii*, showing the different shell layers. C. Detail of the upper part of the prismatic layer of *N. lamarckii*. The individual prisms are separated by thick periostracal membranes. D, E. Views of the external shell surface of *N. lamarckii*. The periostracal surface is ornated with nanometric pimples, which continue on top of the bosses. The bosses appear in high relief due to retraction of the periostracum upon dehydration. F. View of the free periostracum of *N. gemma* showing the regular distribution of bosses. G. Detail of the free periostracum of *N. gemma*. Etching of bosses reveals that they are composed of crystalline sectors. H. Surface of the periostracum of *N. margaritacea*, with cropping out bosses. Its position in J is indicated. I. Detail of the free periostracum of *N. gemma*. Intensive etching reveals lineations within bosses. J. Surface of the periostracum of *N. margaritacea*. Note the existence of bosses within the periostracal flap. K. Surface of the shell of *N. lamarckii*. The outlines of the prisms underlying the periostracum are clearly noticeable. Note the strict correspondence between each prism and its associated boss. L. Similar situation close to a rib of *N. lamarckii*. There appear small-sized additional prisms, which do not bear a boss. M. Fracture through the shell of *N. margaritacea*. The fractured prisms show a typical fibrous aspect. N. Fracture through the columnar nacre of *N. margaritacea*, close to the external surface. O. Fracture through the sheet nacre of *N. margaritacea*, at the mid shell thickness. B, boss; GD, growth direction; N, nacre; Pr, prismatic layer; Po, periostracum.

The shell microstructure for all species examined conformed to the description for all members of the superfamily [7]. The calcareous part of the relatively thick shell is wholly composed of prismato-nacreous aragonite (Fig. 2A, B) [7]. The periostracum is relatively thick (4–7 µm) and persistent, and its continuation can be traced some way down (several tens of microns) into the calcareous part of the shell and forms a series of thick organic sheaths which surround the upper portion of the underlying prismatic layer (Fig. 2C, D). In shells with excellent surface condition, the outer periostracal surface is adorned with pimple-like nanometric, elongate elevations (Fig. 2D, E). More frequently observed, the external surface of the periostracum is studded with regularly spaced crystalline units (∼ 5 µm in diameter and <1 µm in height) (Fig. 2D–I), the bosses described in [7], which sometimes preserve a thin organic cover (Fig. 2D, E) and are totally embedded within the fully grown periostracum (measured thicknesses of 2–4 µm) (Fig. 2C, D, H, J) which wrinkles around their edges (Fig. 2F, H, I). These bosses have polygonal (pseudo-hexagonal) outlines (Fig. 2E–G) and, as previously described [7], [53], represent the initial calcification centres of the prisms of the underlying prismatic layer (Fig. 2B–D, J, K). At lower magnification it is apparent that these crystalline units tend to have a regular distribution in comarginal rows (Fig. 2B, D, F, H–K). The distance between units in a row is more or less the same as between rows and they are completely offset with respect to those of the preceding and succeeding rows, such that the final arrangement of crystalline units is an approximately grid-like reticulate arrangement, with the diagonals along the radial and comarginal directions.

**Figure 3:**
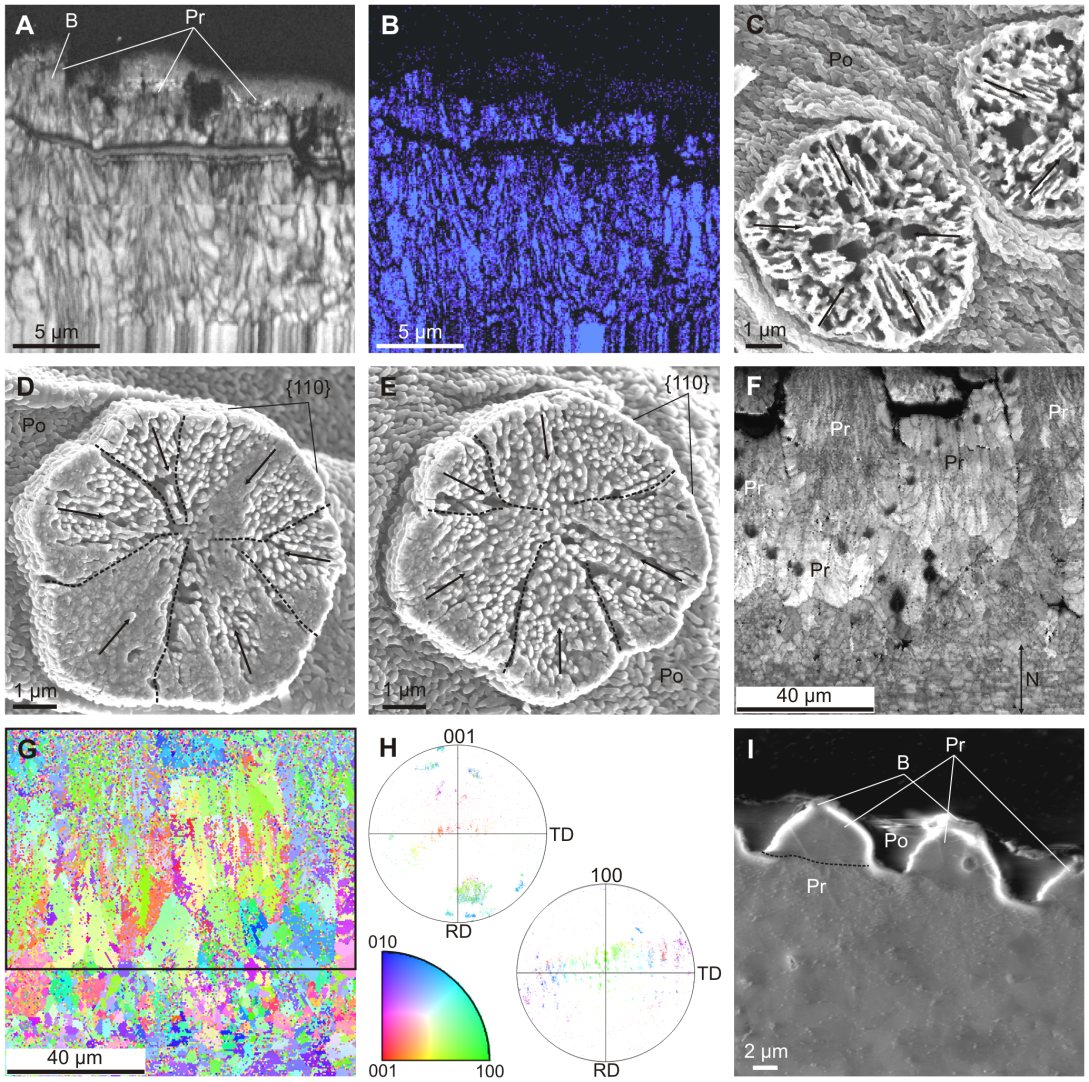
Crystallography of the prismatic units forming the outer layer of *Neotrigonia*. A, B. Image quality and phase map, respectively, of a section through the outer prismatic layer of *N. margaritacea*. The blue color in E indicates that all the mapped material is aragonite, including part of the boss of the left prism. C, D, E. Surface views of bosses of *N. gemma*. The orientations of the lineations revealed upon natural etching have been indicated with arrows in all cases; the peripheral {110} faces, as well as the approximate boundaries between crystals (broken lines) have been indicated in D and E. F, G. Image quality and cleaned up orientation maps, respectively, of another section through the outer prismatic layer and nacre of *N. margaritacea*. H. 001 and 100 pole figures of the framed area in G, and color key (bottom) for the orientation map of G. The 001 pole distribution implies that the *c*-axes of the fibres are mostly parallel to the axes of the prisms, while the 100 pole distribution shows no preferential alignment either within or between prisms. I. Secondary electron image of a polished section through the outer prismatic layer of *N. margaritacea*. The diffraction contrast reveals the difference in texture between the upper part of the prisms (that enveloped by organic periostracal membranes) and the rest of the prisms. The approximate boundary has been delineated in the left prism. B, boss; Po, periostracum; Pr, prism.

The outer prismatic layer consists of aragonitic columnar non-denticular composite prisms which are between 15–30 µm wide and 60–100 µm long (Fig. 2A–D) and which, in longitudinal section, are shown to be composed of fibres radiating from the prism axis (Fig. 2M). There is a strict one-to-one correspondence between bosses and prisms (e.g., Fig. 2K), except at some ornament features, such as knobs and ribs, where some small prismatic units appear to lack the boss (Fig. 2L). The bosses are fully continuous with the prisms with the transition usually taking place smoothly, through a gentle continuous cone-like enlargement of the boss, which later accelerates its widening at the transition to the prism to produce a shoulder (Fig. 2D). In some cases, however, there is a more or less marked constriction at the transition (Fig. 2C). The whole range of transitions can be found within a single species (e.g., *N. lamarckii*; Fig. 2C, D).

The prisms are underlain by nacre (Fig. 2A, B, M). The one at the external half thickness is columnar ( = lenticular, [7]) (Fig. 2N), whereas it is of the sheet type internally (Fig. 2O).

### Mineralogy and Crystallography

The mineralogy and crystallography have been assessed with EBSD. In all cases analysed of *N. margaritacea*, the phase maps (Fig. 3A–B) indicated that the prisms and their bosses are entirely made of aragonite. Orientation mapping of the bosses and the topmost part of the prisms was hindered by the fact that the top part of the prisms (including the bosses) was directly in contact with the embedding resin, which produced differential polishing and, as a consequence, the quality of the diffraction patterns was too low quality for a correct indexation. Nevertheless, some insight can be gained from the surface features of the bosses. In most of them there is some degree of natural abrasion and or etching. This reveals that each boss consists of several radially arranged sectors (Figs. 2F, G, 3C–E). Each sector displays a series of parallel lineations at angles of ∼60° or 120° with those of adjacent sectors (Fig. 3C–E). These lineations have also been revealed in the nacre of bivalves and gastropods [55], [56], in the foliated aragonite of monoplacophorans [57] and in the prismatic aragonitic spikes of anomalodesmatan bivalves [34] and have been recently demonstrated by TEM [58] and EBSD [59] to be parallel to the *a*-axis of aragonite. This therefore suggests that the bosses in *Neotrigonia* are short prismatic units of aragonite consisting of several crystals (sectors) which are polycyclically twinned on {110}. This is consistent with the angles between lineations of adjacent sectors (Fig. 3C–E).

The EBSD maps made on the more internal part of the prismatic units, where they are in coalescence, indicates that each prismatic unit is composed of tens of fibrous units which run more or less parallel to the long axis of the prism and tend to diverge outwards towards the periphery of the prism (Fig. 3A, B, F, G). In section, these units range in width from 1 to 7 µm. Adjacent fibres in a single prism have different colors in orientation maps (Fig. 3G), which implies that they are independent crystals. Although there is a varied array of orientations, most of the fibres come in blue and green colors, which, according to the color key (Fig. 3H, bottom), implies that the 010 and 100 orientations are perpendicular or at a high angle to the section plane (which approximately contains the long axes of the prisms). This is confirmed by examination of the 001 and 100 pole figures (Fig. 3H). The 001 maxima (i.e. the *c*-axes) are aligned with the long axes of the prisms, whereas the 100 maxima are scattered with an approximate equatorial distribution, which implies that the *a*-axes show no preferential orientation.

Although we do not know exactly the position where the prismatic unit changes from being a prism composed of several twinned crystals to a fibrous aggregate, the SEM images obtained under some diffraction contrast show a drastic change at the level where the thick organic membranes disappear (Fig. 3I), which suggests that this is the boundary between the two crystallographic arrangements.

### Formation and Growth of the Periostracum and Bosses

It is evident that the crystalline bosses form *within* the periostracum and indeed they develop within folds of the periostracum even where it is not underlain by mineralized shell (Fig. 2J). The implication of this is that they must develop at an early stage of periostracum formation, necessitating a detailed investigation of periostracal formation in *Neotrigonia* in order to unravel the processes involved. By far, the best results for mantle fixation were achieved in the glutaraldehyde-fixed specimen of *N. margaritacea*, on which most observations on initial formation of the periostracum are based. However in most respects the observations made on the sub-optimally fixed *N. lamarckii* and *N. gemma* were in agreement, except for some details of the mantle lobes (see below). The excellent fixation of our glutaraldehyde-fixed material permits a detailed description of the histology of the periostracal groove and the forming periostracum.

#### Optical and electron microscopy observations of the mantle and decalcified shell

**Figure 4:**
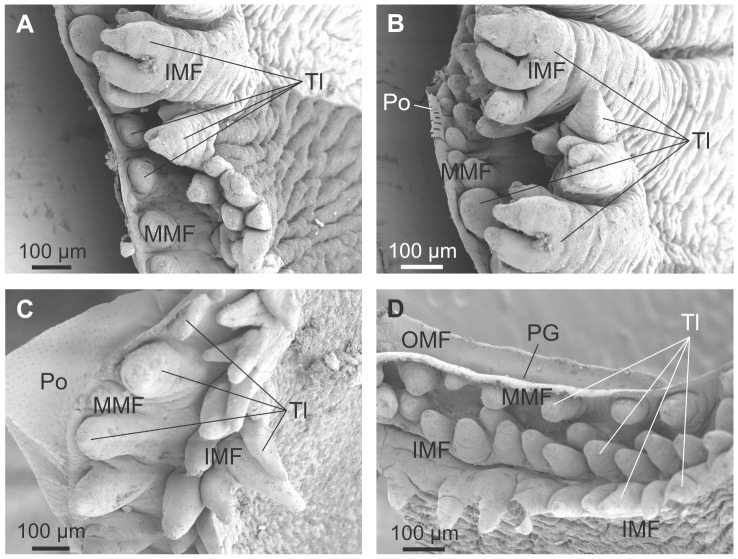
SEM views of the mantle margin of *Neotrigonia margaritacea* and its associated tentacles. A. Mantle around the exhalant siphon. B. Mantle around the inhalant siphon. C, D. Ventral mantle margin. The periostracum can be seen emerging behind the middle mantle fold in C, whereas it is absent in D, thus leaving the internal surface of the outer mantle fold and the periostracal groove exposed. IMF, inner mantle fold; MMF, middle mantle fold; OMF, outer mantle fold; PG, periostracal groove; Po, periostracum; Tl, tentacle.

**Figure 5:**
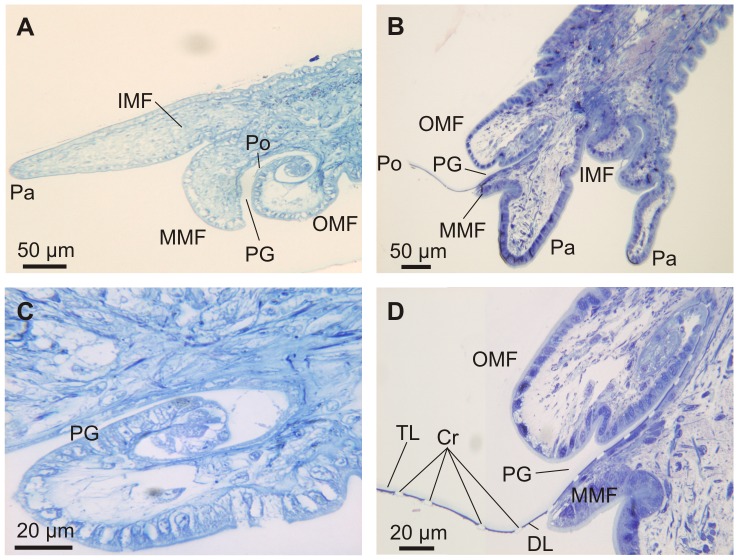
Semi thin sections through the mantle edge of *Neotrigonia* and details of the periostracal groove. A, C. *Neotrigonia gemma*. B, D. *Neotrigonia margaritacea*. Cr, forming crystals; DL, dark layer; IMF, inner mantle fold; MMF, middle mantle fold; OMF, outer mantle fold; Pa, pallial tentacle; PG, periostracal groove; Po, periostracum; TL, translucent layer.

In all three species examined, the outer mantle fold (OMF) is the smallest of the three mantle folds, with the periostracal groove (PG) located between the OMF and the middle mantle fold (MMF). This is evident in both SEM (Fig. 4) and OM (Fig. 5) views. SEM of the mantle margin of *Neotrigonia margaritacea* reveals that it is provided with three rows of densely distributed tentacles all along its length, two of the rows settling onto the IMF and the remaining one onto the MMF. Tentacles of the IMF are particularly developed around the area of the siphons, where they even develop branched tips (Fig. 4A, B). In some views, the periostracum can be seen emerging from the PG (Fig. 4B, C). Optical microscopy views are drastically different depending of the number and position of sectioned tentacles (Fig. 5A, B). In all cases, the periostracal groove coils inwards towards the interior of the OMF for more than one and a half turns (Fig. 5C, D) with a flattened spiral shape elongated in the direction of the shell formation.

**Figure 6:**
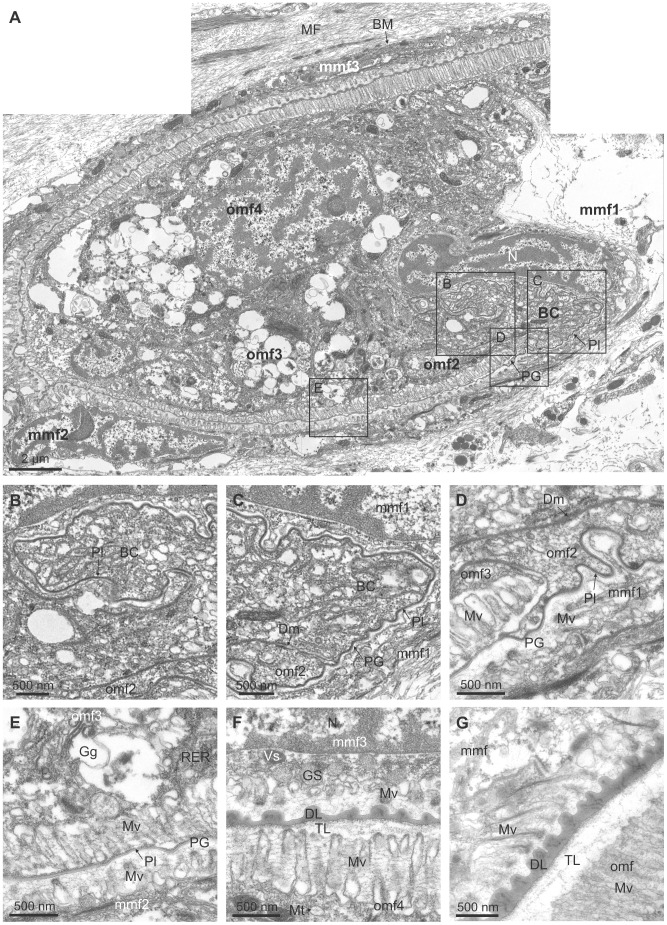
TEM views of the periostracal groove area of *Neotrigonia margaritacea*. A. General view of the initiation of the periostracal groove and the basal cell. The positions of B to E are indicated. B. Pellicle forming at the basal cell. C. Aspect of the pellicle immediate before the initiation of the periostracal groove. D. Initiation of the periostracal groove. E. Aspect of the periostracal groove one quarter of a whorl after its initiation. The pellicle has not yet thickened appreciably. F. Aspect of the periostracal groove, one whorl after its initiation. This position is slightly outside the view shown in A. The periostracum has thickened appreciably and the surface looking towards the middle mantle fold is ornamented with dimples. G. Aspect of the periostracum at a more advanced stage. The dark layer has coarsened and an underlying translucent layer is evident. BC, basal cell; BM, basal membrane; DL, dark layer; Dm, desmosome; Gg, Golgi complex; GS, granules of secretion; Mt, mitochondrion; MF, muscular fibres; mmf, cell of the middle mantle fold; mmf1, mmf2, mmf3, middle mantle fold cells 1, 2 and 3; Mv, microvilli; N, nucleus; omf, cell of the outer mantle fold; omf2, omf3, omf4, outer mantle fold cells 2, 3 and 4; PG, periostracal groove; Pl, pellicle; RER, granular endoplasmic reticulum; Vs, electron-dense vesicle; TL, translucent layer.

The periostracum originates in the basal cell (Fig. 6) (which can be considered as the first cell of the OMF) at the base of the coiled PG (Fig. 6A–C) and is extruded as a thin, irregularly folded pellicle of *ca*. 25 nm thickness, into the narrow space between the basal cell and the first cell of the MMF (labelled mmf1 in Fig. 6A) (Fig. 6C, D). This interspace may be considered as the start of the periostracal groove. The elongate basal cell is characterised by an absence of microvilli and the presence, within the cytoplasm, of a Golgi complex with many electron-dense and electron-lucent vesicles, abundant granular endoplasmic reticuli and electron-dense granules, as well as scattered mitochondria (Fig. 6A–C). Intercellular junctions (desmosomes) occur between the basal cell and the second cell of the OMF (omf2 in Fig. 6A) (Fig. 6C, D). This second cell of the OMF has also an elongated shape, due to the spiral turn of the OMF, and a small free surface without microvilli (Fig. 6D). Its cytoplasm resembles that of the basal cell and is connected to the next epithelial cell by desmosomes. The third cell of the OMF (omf3 in Fig. 6A) (Fig. 6A, E) has an elongated microvillous free surface, which is prolonged on the second cell, forming an apical process to which the periostracum is tightly attached as it leaves the basal cell (Fig. 6D). This third cell contains many large electron-lucent vesicles (100–200 nm in diameter), concentrated at the apical region near the microvilli (Fig. 6E). Many horizontal nano-fibrils are associated with this cell, in the interspaces between the microvilli (Fig. 6E). The fourth cell of the OMF has its free microvillous surface opposed to the third cell of the middle mantle fold (omf4 and mmf3 in Fig. 6A and F). This elongate cell also has a well-developed Golgi complex, with many electron-lucent vesicles.

**Figure 7:**
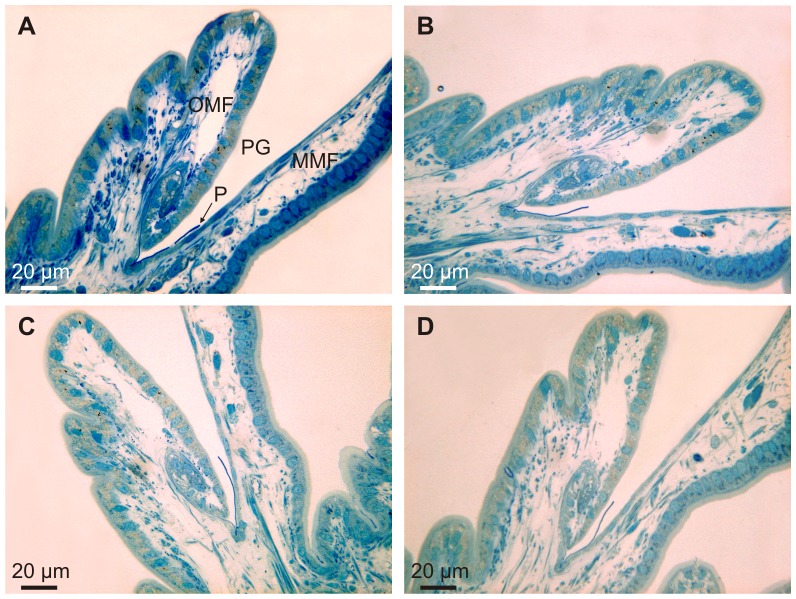
Semi-thin section of the periostracal groove area of *Neotrigonia margaritacea* stained with different toluidine Blue (TB) solutions. A. Greenish metachromasia of the epithelial glands of the outer mantle fold after staining with with 1% aqueous TB and sodium tetraborate (1%). B. Brown-greenish metachromasia with 1% aqueous TB and acetic acid (0.2%). C. Brown-greenish metachromasia with 0.5% aqueous TB and acetic acid (0.2%). D. Brown-greenish metachromasia obtained with 0.2% aqueous TB and acetic acid (0.2%). MMF, middle mantle fold; P, periostracum; PG, periostracal groove; OMF, outer mantle fold.

During the first half turn of the PG, the periostracum does not appreciably increase in thickness but becomes folded, with variable wavelengths of several hundreds of nm (Fig. 6A, E). Half a turn later along the PG, the periostracum begins to increase in thickness (to 70–80 nm) and the folds become sealed at their bases by additional secretions from the microvilli of the cells of the inner surface of the OMF (Fig. 6F). These secretions contribute to form the dark layer (DL) of the periostracum. An incipient translucent layer (TL) (of the type found in anomalodesmatans and other bivalves; see references in [34]) is discernible below the DL at this point (Fig. 6F). From the next half turn onwards, there is a clear increase in thickness of both the DL and TL (which is clearly laminated on a nanometric scale) (Fig. 6G). From this point to the end of the PG, the periostracum adheres to the outer surface of the MMF (Fig. 5B, D) and over this course the thicknesses of both the DL and TL increase steadily to a position which is approximately coincident with the tip of the OMF (Fig. 5D). Along this portion of the PG, the inner side of the OMF consists of a single layer of epithelial, tall, columnar cells with microvilli on the free surface (Fig. 5C, D, 7). The nuclei of these cells are nearly central and elongate perpendicular to the free surface; there are abundant glandular vesicles in their cytoplasm. The 1% aqueous toluidine blue gave a greenish metachromasia with the addition of 1% Na_2_B_4_O_7_.10H_2_O at pH 4.5 (Fig. 7A), but changed to brown-greenish when 0.2% acetic acid was added instead to any of the toluidine blue concentrations (Fig. 7B–D). These glandular vesicles are more numerous in the distal half of the OMF and continue along its outer side. The microvilli of the OMF epithelial cells are long and show marked secretions of pinocitic vesicles and small fibrils thereby thickening the TL of the periostracum (Fig. 6G). The outer surface of the MMF is a monolayer of squamous cells, with nuclei elongated and parallel to their microvillous free surface (Fig. 7). The cytoplasm of these cells is rich in vesicles with different electron densities, but do not have glandular vesicles of the type present in the OMF (Fig. 7). Under this cell layer, there is a basal membrane and muscular fibres (Fig. 7).

**Figure 8:**
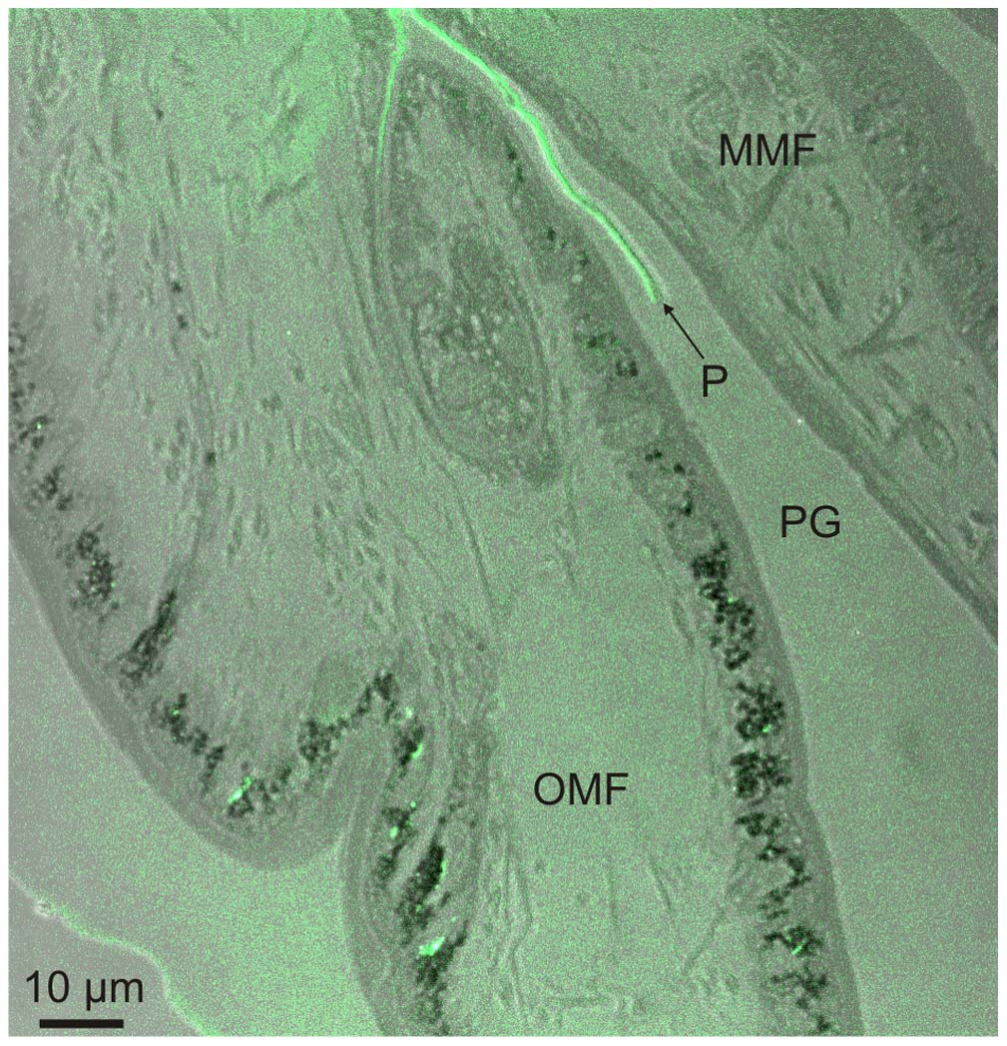
Confocal laser image of the periostracal groove and outer mantle fold area of *Neotrigonia margaritacea*. The green fluorescent colour present in the epithelial cells of the outer mantle fold and in the forming periostracum points to the presence of Ca^2+^ ions that are bound to the fluorescent indicator. MMF, middle mantle fold; P, periostracum; PG, periostracal groove; OMF, outer mantle fold.

The observation under confocal microscope of semi-thin sections treated with fluorescent indicator reveals a high fluorescence signal both in the forming periostracum as well as in the interiors of the cells lining the internal and external surfaces of the outer mantle fold (Fig. 8).

**Figure 9:**
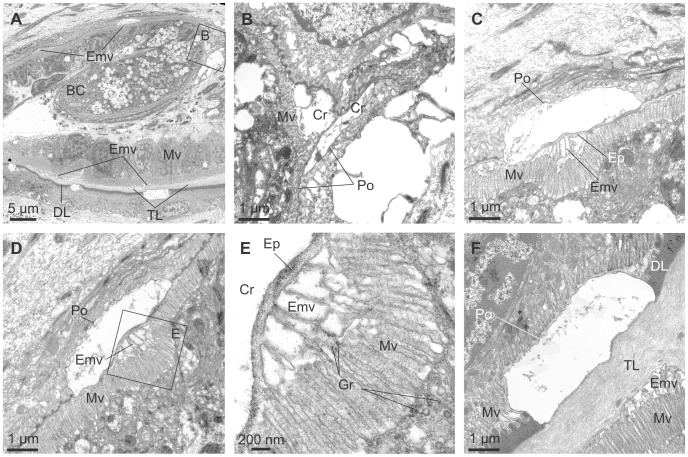
Crystallization within the periostracal groove of *Neotrigonia margaritacea*. A. General view of the periostracal groove area, showing the forming periostracum and incipient aragonite crystals. B. Forming crystals at about half a whorl after the initiation of the periostracal groove. Its position in A is indicated. C, D. Forming crystals at about three quarters of a whorl. The periostracal cover is much thinner than the surrounding periostracum. A thin translucent layer is visible below the dark layer. E. Detail of the reticulate extensions of the microvilli developed below the forming crystal shown in D (indicated). F. Crystal at two and a quarter whorls along the periostracal groove. There are thick dark and translucent layers. BC, basal cell; Cr, crystal; DL, dark layer; Ep, Extrapallial space; Gr, electron dense granules; Emv, reticulate extensions of the microvilli; Mv, microvilli; Po, periostracum; TL, translucent layer.

The first calcified structures within the periostracum are observed by TEM at a very early stage of periostracum formation, at the end of the initial first half turn of the PG (Fig. 9A, B). The early initiation of calcification is further supported by fact that all crystals so far examined have only a small thickness of periostracal cover (25–30 nm) (Fig. 9B, C, D, F), which is not significantly different from the thickness of the initial pellicle, thus implying that there is no crystal secretion after this initial half turn, by which time the thickness of the dark layer is far greater. As the crystals progress along the PG their height increases (compare Fig. 9C, D to F). Before the TL clearly develops, crystals seem to be directly in contact with the microvilli of the cells lining the inner surface of the outer mantle fold (Fig. 9C, D), although we lack the resolution to reject firmly the possibility that a nanometric TL might be present at this point. It is of note that the growth surfaces of the crystal plaques always remain at the transition between the TL and DL.

Before the TL develops appreciably, there is a narrow extrapallial space (∼100 nm) extending between each growing crystal and the microvilli of the underlying OMF cells (Fig. 9C–E). The microvilli extend between the crystal growth surface (to which they conform tightly) and their cells. Many electron-dense granules are observed in the cytoplasm, between and within the microvilli (Fig. 9E). In the cytoplasm of each of the cells placed under the crystal there is a well-developed Golgi complex, formed by a pillar-like curved vesicles (Fig. 9A, C–E). When the TL becomes conspicuous, it separates the crystals from the microvilli (Fig. 9F). The microvilli develop closed columnar to vesicular formations at the positions of the crystals below the TL (Fig. 9A, F). These columnar formations receive the secretion of numerous Golgi vesicles throughout the microvillar border. From here on, we will refer to these structures derived from the Golgi secretions as reticulate extensions of the microvilli (Emv in Fig. 9). The height of these early crystals grows with the thickness of the DL; hence their growth surface keeps pace with the boundary between the DL and the TL.

**Figure 10:**
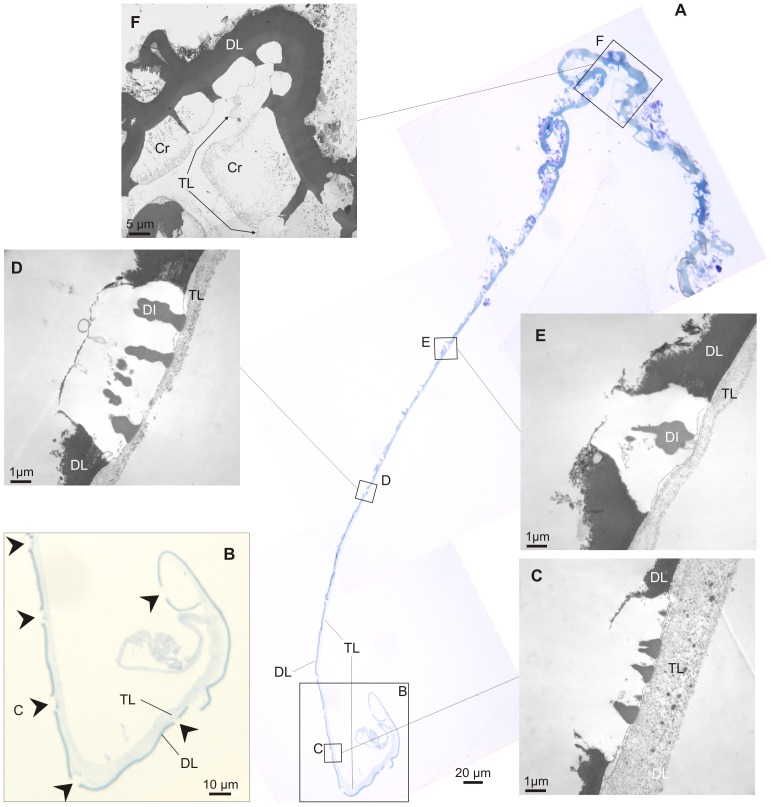
Crystal evolution along the periostracum and shell margin of *Neotrigonia gemma*. A. Semi-thin section of the decalcified periostracum and shell margin. B. Enlarged view of the initial part, to show the relative thicknesses of the dark and translucent layers. The translucent layer has detached from the dark layer at the initialmost portion. Arrows point to positions where crystals have been dissolved. C–F. TEM views of forming crystals at progressively older positions. Along the age series the crystals increase in height. The growth surface stays always at the boundary between the dark and translucent layers. Note relative thickness of the translucent layer in C and remains of translucent layer between crystals in F. Cr, decalcified crystals; DI, dark inclusions; DL, dark layer; TL, translucent layer.

Fig. 10A shows a portion of decalcified periostracum detached from the PG. The spiral shape of the PG is mirrored in the earliest part of the periostracum (Fig. 10B), and, consistent with this, shows a relatively thick TL at this position. TEM observations of this same periostracum and forming crystals show that both the DL and crystals increase in height in a coordinated manner such that the growth surface of crystals keeps pace with the DL-TL transition (Fig. 10C–E). In all cases, crystals show inclusions of a material similar to the dark layer which are more or less perpendicular to the growth surface and which appear at some depth within the crystals. These structures are never observed in the top portions of the crystals (i.e. those forming deep inside the PG) (Fig. 10C–E), which suggests that they developed later, possibly by absorption of tanned parts of the TL. At the shell edge, growing units attain their full width and begin to impinge on each other, but they are still separated by organic walls (2–3 µm thick) which are clearly extensions of the DL and which still retain remains of the TL at their bases (Fig. 10F).

#### SEM observations of the shell

**Figure 11:**
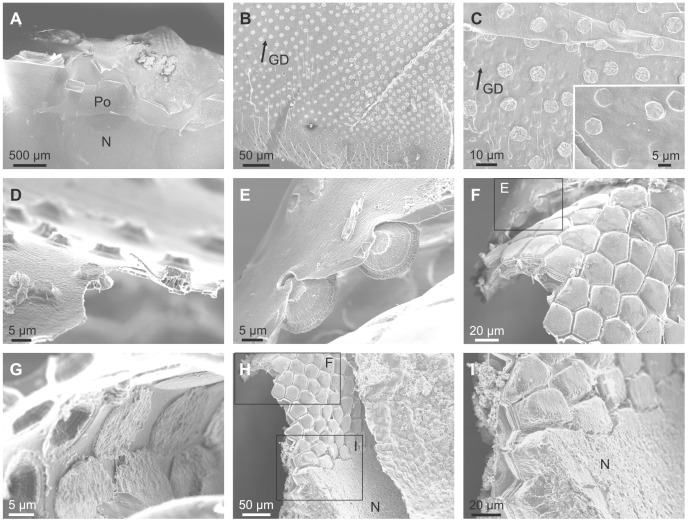
SEM views of the periostracum and shell margin of *Neotrigonia gemma*. A. General view of the shell margin. The periostracum has adhered to the internal shell surface. B. Initial part of the periostracum. In this portion, the bosses increase in diameter in the growth direction. C. Detail of the initial bosses, some of which clearly retain the initial periostracal cover (inset). D. Aspect of the forming crystals half way to the shell margin showing that the initial bosses had grown into truncated conical crystals. E. Aspect of the crystals close to the shell margin. The position in F is indicated. F. General aspect of the folded shell margin. The position in H is indicated. Along the fold the conical crystals shown in E transform into short prisms separated by periostracal membranes. G. Detail of the interior surface of the fold. The bases of the crystals have expanded to a maximum and are about to impinge with each other. H. A general view the shell margin, which includes the completion of the prismatic layer and the transition to nacre. I. Detail of the transition prisms-nacre. The prisms are separated by gaps, which are clogged with crystalline material upon the onset of nacre secretion. The position in H is indicated. GD, growth direction; N, nacre; Po, periostracum.

Examination of specimens in which the periostracum has detached from the PG (Fig. 11A) reveals that it is studded from its very initiation (i.e., in areas most probably within the PG) with evenly spaced pseudo-hexagonal tablets of aragonite (Fig. 11B), which are sometimes covered by a thin frilled pellicle (Fig. 11C). In one sample in which the free periostracum has detached from the associated mantle, it can be seen how the initial plaques or bosses (or the hollows left by them) initially grow in width towards older areas until a constant size is finally attained (Fig. 11B). As suggested by the TEM data, this process must takes place within the PG. During the transition towards the shell edge, the bosses progressively grow in height and expand at their bases thereby acquiring a conical truncated aspect (Fig. 11D, E). The process of growth and expansion of the forming crystals continues up to the shell edge at which point the periostracum is reflected back onto the outer surface of the mantle (Fig. 11F). Here, growing units attain their full width (Fig. 11G) and begin to impinge with each other, thus acquiring a prismatic outline (Fig. 11F, H). In their early stages, prisms are still separated by organic walls (2–3 µm thick) (Fig. 11F) which extend some way within the shell interior (from 6–7 µm in *N. lamarckii* to around 20 µm in *N. margaritacea*), after which the aragonitic prisms continue to grow to the completion of the full thickness of the outer prismatic aragonitic layer (Fig. 11H, I). After the disappearance of the dark organic walls, the prisms continue to grow by keeping a gap more or less equivalent to the thickness of the walls (Fig. 11I). When the prisms have attained their full height, the nacreous layer is secreted below. In coincidence with the onset of the secretion of the (rapidly thickening) nacreous layer, the spaces between prisms are completely filled with additional material (Fig. 11I). In this way, the prismatic layer transforms into a compact material and a coherent shell layer.

## Discussion

### Formation of the Periostracum and Bosses in *Neotrigonia*

**Figure 12:**
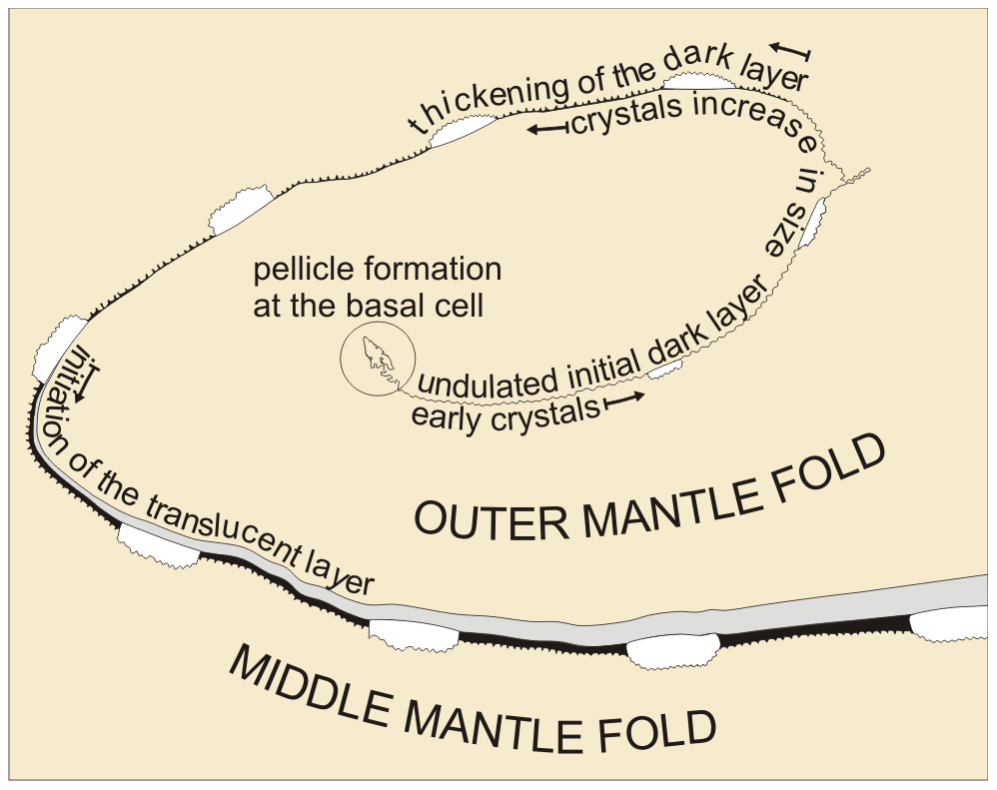
Schematic diagram depicting the main events observed along the periostracal groove in *Neotrigonia*.

**Figure 13:**
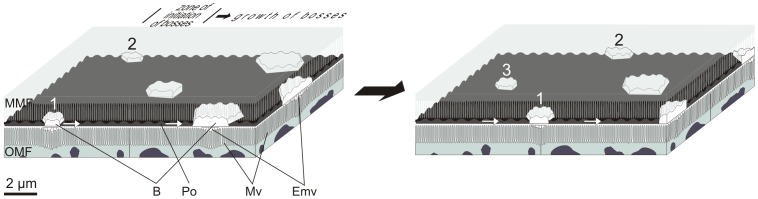
Schematic diagram depicting the initiation and early growth of bosses within the periostracal groove. Bosses inititate at subcellular positions which are equally spaced comarginally along the outer mantle fold. Growth proceeds as the periostracum slides between the mantle folds by secretions of the outer mantle fold. Below each boss there is a reticulum formed by extensions of the underlying microvilli, which also migrates across the cells in coincidence with the boss. The time of initiation alternates between rows, with the formation of bosses labelled 1 and 2 in the left diagram and of boss 3 in the right diagram. B, boss; Emv, reticulate extensions of the microvilli; MMF, middle mantle fold; Mv, microvilli; OMF, outer mantle fold; Po, periostracum.

Our observations for the origin in the basal cell and extrusion of the periostracum along the PG can be summarised and interpreted as follows, with reference to Fig. 12:

1 *Neotrigonia* has a distinctive spiralling periostracal groove.
2 The periostracum, secreted by the basal cell, is initially no more than a thin pellicle (25–30 nm thick). During the first half turn of the PG there is no appreciable thickening of this layer but it does become folded (Fig. 6A, E). At the same time, the first crystal plaques form below the initial periostracum (which, at this stage, seems to be constituted by the pellicle) (Fig. 9A, B). Given the grid-like reticulate arrangement of bosses, there must be particular areas of the outer mantle surface where cells are specialized for the production of new bosses and which are evenly spaced along the comarginal direction (positions 1 and 2 in Fig. 13). Once a new boss has been produced, the production subcellular site enters a refractory period until it becomes again activated for the production of a new boss. In this way, the bosses become regularly spaced in the longitudinal direction. For the grid-like pattern to become complete intermediate production sites (position 3 in Fig. 13) should become activated alternatively. The observed reticular extensions of the microvilli underlying the forming crystals (Fig. 9A, C–F) must, therefore, migrate in coincidence with these (Fig. 13). This model implies that the production of bosses is under genetic control.
3 The bosses acquire their full width very soon within the early coiled portion of the PG. Their patterning into sectors observed upon etching (Fig. 2E, F, G) allows us to determine that tablets are in fact polycyclically twinned crystals (Fig. 3C–E). By comparison with other similar instances of biogenic aragonite (e.g., spikes of anomalodesmatans [34]) we can infer that the *c*-axis is perpendicular to the surface of the plaque and that the *a*-axes run in parallel to the observed lineations.
4 From the beginning of the second half turn towards the exit of the PG, the folds of the DL become united by additions of new material by the cellular microvilli (Fig. 6F). The observed huge reticulate extensions of the microvilli developed by the OMF epithelial cells below each forming crystal are produced by active secretions of vesicles from the Golgi complex throughout the microvilli (Fig. 9C–E). This points to an active transport of calcium (either in the form of ions or as amorphous calcium carbonate [60]) and to the presence of organic compounds (such as phenolic-acid mucopolysaccharide complexes and sulfated glycoproteins). The application of fluorescent indicator fluo 3 AM on semithin sections has shown the presence of Ca^2+^ both in the epithelial cells of the outer mantle fold and in the forming periostracum (Fig. 8). Due to lack of resolution we could not test under the confocal the likely presence of Ca^2+^ in the vesicles opening to the reticulate extensions of microvilli seen under the TEM (Emv in Fig. 9). Jaiswal et al. [61] found that membrane proximal lysosomes are the major vesicles responsible for calcium-dependent exocytosis in nonsecretory cells.

Toluidine blue is known to cause red and purple metachromasia with acid mucopolysaccharide and glycoprotein respectively. Green metachromasia by toluidine blue has been reported, among other instances, in the organic cups of the solenogaster *Proneomenia agalaophaeriae* and inner cuticle of the chiton *Acanthochitona crinitus* [62], and in the vitelline cells of monogenetic platyhelminthes *Pricea* and *Protomicrocotyle*, and freshly moult epicuticle of the millipedes *Spirostreptus asthenus* [63] and these authors have shown that this is related to the presence of diphenols. Histochemical examination of the cuticle of *Proneomenia* showed that the matrix was composed of a glycoprotein complex with highly acidic mucopolysaccharide and low protein contents in which tanning plays little part in stabilization [62]. These authors considered that the cuticle of the Aplacophora is tentatively equated with an early mucoid stage in the evolution of the molluscan shell and it is suggested that secretion of additional protein, followed by hardening by quinone-tanning, are necessary stages before a calcified shell evolved. The presence of phenolic mucopolysaccharide complexes in the epithelial cells of the outer mantle fold of *Neotrigonia margaritacea* seems to point to a more intense hardening of the periostracum. The brownish metachromasia is usually related to the presence of sulfatide and acidic lipids [64]. In the epithelial cells of *Neotrigonia margaritacea*, the brownish metachromasia could be due to the link to sulfated glycoproteins with toluidine when acetic acid is added to the aqueous solution. Taking the above into account, the epithelial glands of the outer mantle fold of *N. margaritacea* may contain a phenolic-acid mucopolysaccharide complex and sulfated glycoproteins, among other secretions, which would be related both to the formation of the organic matrix and hardening of the periostracum.

The distance between the crystals and the tips of the microvilli or of their reticulate extensions (estimated at ∼100 nm) is the so-called extrapallial space, e.g. [14], [65] (Ep in Fig. 9C–E). Since the crystals are completely enclosed within the mantle tissues and in view of the consistency of the estimated thickness, we are confident that the extrapallial space is imaged with its correct dimension. Both the reticulate extensions of the microvilli and the extrapallial space are imaged here for the first time in molluscs.

5 From the third half turn of the periostracal groove onwards, a conspicuous TL begins to appear and rapidly increases in thickness, so as to constitute the bulk of the periostracum (Fig. 9A, F). The DL also thickens probably due to tanning and condensation of the TL, as observed in other bivalves [66]. The crystalline bosses increase in thickness, by keeping pace with the DL, at the same time that they expand in width.
6 During the free periostracum stage, i.e. that portion of the periostracum which spans the gap between the PG and the shell edge, the periostracum slides along the surface of the OMF at the same time that both periostracal layers thicken (Fig. 5D). The TL thickens by secretions of the OMF, whereas the DL thickens by tanning of the TL. When the periostracum leaves the tip of the OMF, the TL ceases to be secreted and its thickness begins to decrease due to continued transformation by tanning into DL. The presence of large and numerous glandular vesicles in the cells of the OMF, which stain greenish or brownish with toluidine blue (Fig. 7), may be related to the secretion of phenolic-acid mucopolysaccharide complex and sulfated glycoproteins that contribute to the formation of the organic matrix and to the tanning process of the TL into DL layer by phenolic secretions.

At the same time, the crystalline bosses continue to thicken and expand until they impinge upon each other acquiring a prismatic morphology (Fig. 11C–H). During growth of the prismatic layer, the prismatic units still continue to be separated by thick organic walls made of DL, which are formed at the expense of the last residues of TL (Figs. 10F, 11H).

7 The prisms continue to increase in length beyond the periostracal walls, while still being separated by intervening spaces. At this time each prism has been transformed into a polycrystalline aggregate of longitudinal fibres (Fig. 3D–G).
8 Finally, the nacreous layer begins to be secreted below the prismatic layer (Fig. 11H). At this point, the spaces between prisms become filled with additional mineral material (Fig. 11I).

### Comparison of the Formation of the Periostracum and Initial Crystals in *Neotrigonia* with Other Bivalves

The architecture of the mantle edge of *Neotrigonia* is similar to that which has been described for other bivalves. As previously noted [50], there are three, relatively unspecialised mantle folds which may be described using the standard nomenclature of Yonge [67], [68], although there is some morphological variation between species. The presence of tentacles in the middle and inner mantle folds is a general feature in the genus. Although not remarked upon by Morton [50], it is apparent from his figure 2 that his material of *N. margaritacea* also had a coiled periostracal groove. The presence of a spiral-shaped periostracal groove is unusual but has also been reported in the heterodont cardiids *Cerastoderma edule* [69] and *Acanthocardia tuberculata* (unpublished data), as well as in the unionoids *Potomida littoralis* [24] and *Unio pictorum* (pers. obs.), the sister group to the trigonoids [47]. In all these cases, the periostracum also coils towards the OMF, but only for less than half a turn. As in other studies [66], [70]–[74], the periostracum originates from a basal cell. Although a few studies on, e.g. *Nucula sulcata* [73] and *Cerastoderma edule* [69], have indicated that some bivalves have two basal cells, we have no positive evidence that this is also true for *Neotrigonia.* The epithelial cells of the MMF fold are columnar in some bivalves species [71] or cuboidal in most of them [72], [75], but in *Neotrigonia* they are squamous and very stretched out. The cells lining the inner surface of the OMF are columnar and contribute to the formation of the TL of the periostracum, as in most of the studied bivalves [66], [69], [74], [75] (Figs. 5, 7 and 9A).

Our results show that the initial crystalline plaques which constitute the bosses of the prisms and the external part of the outer prismatic layer are entirely intraperiostracal. The previous suggestions that the bosses pierce the outer surface of the periostracum [36] are mistaken, based on imperfectly preserved material where the ultra-thin periostracum covering (in fact, the pellicle) has been lost.

**Figure 14:**
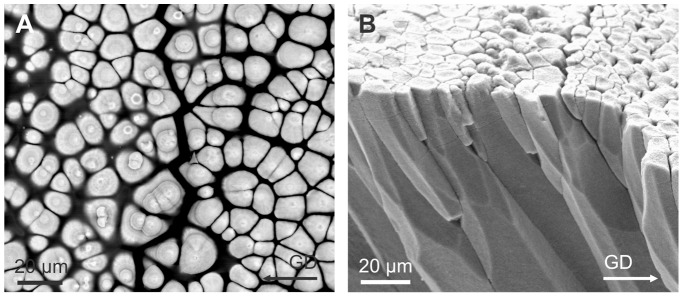
Outer aragonitic prismatic layer of Unionidae. A. View of the shell surface of *Unio elongatulus*, in which the periostracum has been removed with bleach. The distribution and size of the prisms is irregular. Some prisms have bosses, whereas others have not, which implies that they initiate at different positions within the periostracum. B. Bleached fracture of the shell of *Lamprotula* sp. The prisms show big differences in size and density, and many of them disappear towards the shell interior (bottom direction), due to selection by competition. GD, growth direction.

The form of the bosses in *Neotrigonia* is very similar to those seen in the anomalodesmatans and unionoids, perhaps indicating some homology. There are, however, several characteristics of spike formation that are exclusive to *Neotrigonia*. Of particular note is that our observations demonstrate the very early onset of calcification within the periostracum of *Neotrigonia,* deep within the periostracal groove. Detailed investigation of periostracal calcification on other Recent bivalves, e.g. the gastrochaenid *Spengleria* [35], [37] and many anomalodesmatans [34], [76], indicate that in those groups these structures are initiated rather later in the process of periostracum formation only at the free periostracum stage, within the TL, at its boundary with the DL, after the sheet has exited the periostracal groove. Re-examination of previously published material [24] and other additional material indicates that the bosses of the prisms of Unionoidae (which, if present, are much less protrusive than those of *Neotrigonia*) form well within the periostracum, most probably only after this has reflected back onto the external surface of the shell. Zieritz et al. [36] also described mineralized spikes in 20 species of Unionoida, which are enclosed very shallowly within the periostracum. Although that study did not include a TEM study of their formation, their appearance on the shell surface [36] is more similar to that of the spikes of anomalodesmatans than to the bosses of *Neotrigonia* in that they remain well within the periostracum. Two additional, related features which are unique to *Neotrigonia*, are (1) the strictly regular distribution of initial bosses and (2) the one-to-one correspondence between bosses and prisms of the outer layer, which, together, imply that the number and position of prisms are determined already within the periostracal groove. When, during growth, bosses transform into prims and occupy all the available space, the resulting sizes and distribution patterns of the prismatic units is highly regular. This is in contrast with what is observed in other aragonitic prismatic layers (e.g., those of their close relatives, the unionoids), in which prisms may differ in the position within the periostracum as well as in size (Fig. 14A). Also the density may easily change from one position to another (Fig. 14A, B). This implies that the formation of unionid prisms are not under such an strict genetic control. These differences are responsible for the high degree of selection by competition of prismatic units [77], [78]) (Fig. 14B), which is totally absent in *Neotrigonia*.

Understanding how calcium carbonate crystallises within an organic layer may have considerable interest outside the realms of molluscan evolution. The ability to secrete a hard shell occurred ‘rapidly’ in disparate metazoan groups as part of the Cambrian explosion [79], [80]. Many of the early biomineralising taxa produced spicules or plates of calcium carbonate, presumably held in an organic sheath (see [81] for a review). Although the sclerites and spicules of many of these early scleritome-bearing organisms are rather complex structures [81], an understanding of how mineralization proceeds within an organic sheet may be instructive in understanding their evolution. Our findings of the very early onset of calcification within the growing periostracal sheet of these bivalves, is perhaps of broader interest as it suggests one model by which biomineralization may proceed from isolated units within an ‘organic’ cuticle, and which subsequently merge to form a recognizable shell.
